# Morphological and genetic evidence support the reinstatement of the long-forgotten *Telipogon teuscheri* (Orchidaceae, Oncidiinae) from southwestern Ecuador

**DOI:** 10.3897/phytokeys.273.180600

**Published:** 2026-04-09

**Authors:** Luis E. Baquero, Carlos Martel, Yanua Ledesma, Mónica Salomé Guerrero-Freire, Gabriel A. Iturralde

**Affiliations:** 1 Grupo de Investigación en Biodiversidad, Medio Ambiente y Salud (BIOMAS), Carrera de Ingeniería en Agroindustria, Facultad de Ingenierías y Ciencias Aplicadas, Universidad de Las Américas, UDLA, Vía a Nayón, Quito 170124, Ecuador Instituto de Ciencias Ómicas y Biotecnología Aplicada, Pontificia Universidad Católica del Perú Lima Peru; 2 Royal Botanic Gardens, Kew, TW9 3AB, Richmond, London, UK Royal Botanic Gardens, Kew London United Kingdom; 3 Instituto de Ciencias Ómicas y Biotecnología Aplicada, Pontificia Universidad Católica del Perú, 15088, Lima, Peru Facultad de Ingenierías y Ciencias Aplicadas, Universidad de Las Américas Quito Ecuador; 4 Programa de Doctorado, Facultad de Ciencias Veterinarias, Universidad de Buenos Aires, Buenos Aires, Argentina Facultad de Ciencias Veterinarias, Universidad de Buenos Aires Buenos Aires Argentina; 5 Colegio de Ciencias de la Salud, Universidad San Francisco de Quito, USFQ, Quito, Ecuador Colegio de Ciencias de la Salud, Universidad San Francisco de Quito Quito Ecuador

**Keywords:** Andes, Azuay Province, epiphytic orchids, molecular phylogenetics, nomenclature, synonymy assessment, taxonomy, Andes, Provincia de Azuay, orquídeas epífitas, filogenia molecular, nomenclatura, taxonomía

## Abstract

*Telipogon teuscheri*, a rare orchid species from southwestern Ecuador, was described by Garay in 1958, but has long been forgotten after being placed in synonymy with *T. tesselatus* by Dodson in 1989. Revision of type material, herbarium specimens, field observations, and collecting itineraries, along with genetic analyses, has revealed that *T. teuscheri* is morphologically and genetically distinct from *T. tesselatus*. Furthermore, our revision of herbarium material and iconography indicates that *T. isabelae*, described by Dodson in 2004, is conspecific with *T. teuscheri* and should be treated as a synonym, thereby resolving a long-standing taxonomic confusion. We present a detailed morphological description, illustrations, a distribution map, the phylogenetic position, and notes on habitat, phenology, and conservation status of *T. teuscheri*.

## Abstract (es)

*Telipogon teuscheri*, una rara especie de orquídea del suroccidente de Ecuador, fue descrita por Garay en 1958, pero ha sido olvidada por largo tiempo después de haber sido sinonimizada con *T. tesselatus* por Dodson en 1989. Revisiones de materiales tipo, registros de herbario, observaciones de campo e itinerarios de colecta, junto con análisis genéticos revelan que *T. teuscheri* es morfológica y genéticamente distinta a *T. tesselatus*. Adicionalmente, nuestra revisión de material de herbario e iconografía indica que *T. isabelae*, descrita por Dodson en 2004, es conespecífica con *T. teuscheri* y debe ser tratada como su sinónimo, resolviendo una confusión taxonómica de larga data. Proveemos una descripción morfológica detallada, ilustraciones, un mapa de distribución, evidencia filogenética y notas sobre el hábitat, la fenología y el estado de conservación para *T. teuscheri*.

## Introduction

The Neotropical genus *Telipogon* Kunth (Orchidaceae, Oncidiinae) comprises ca. 250 species of mostly epiphytic orchids distributed from Mexico to the Andes, with its center of diversity in Colombia, Ecuador, and Peru (Dodson 2004; Chase 2009; POWO 2025). *Telipogon* species are notable for their intricate, often sexually deceptive flowers, characterized by a distinctive, ornamented gynostemium, particularly in the form and distribution of trichomes, and an uncinate viscidium (Martel et al. 2020). Historically, the genus *Stellilabium* Schltr. was treated as distinct from *Telipogon*, but genetic studies supported the transfer of their species within *Telipogon* (Williams et al. 2005; Chase 2009; Neubig et al. 2012). The expanded circumscription now includes both large-flowered and miniature species (Martel et al. 2017).

Calaway H. Dodson (1928–2020) contributed extensively to the understanding of *Telipogon* taxonomy in Ecuador and adjacent countries (e.g. Dodson and Escobar 1987a, 1987b, 1993a, 1993b; Dodson and Dodson 1989; Dodson and Bennett 1989; Dodson 2002, 2004). Among his studies of *Telipogon* from southern Ecuador, he proposed *T. isabelae* Dodson & Hirtz as a distinct species based on a plant collected near Santa Isabel in the Azuay Province (Dodson 2004). He also reduced *Telipogon teuscheri* Garay, from near Cuenca, to a synonym of *T. tesselatus* Lindl., which was based on material near Loja (Dodson and Bennett 1989). The circumscription of *T. teuscheri* as a synonym of *T. tesselatus* has remained accepted by online databases (Missouri Botanical Garden 2025; POWO 2025) and in a recent taxonomic account of *Telipogon* from the northern Andes (Szlachetko et al. 2022). However, to the best of our knowledge, no detailed studies have been carried out to clarify the identity of *T. teuscheri* or *T. isabelae*, both of which have been known from their holotypes only. Recent fieldwork in the type localities, together with the review of the protologues and herbarium material, revealed inconsistencies in the identity of *T. isabelae*, *T. teuscheri* and *T. tesselatus*. This prompted a re-evaluation of these taxa.

Here, using a combined approach of fieldwork, morphological analyses of herbarium material and genetic analyses, we (1) provide evidence that the inclusion of *T. teuscheri* in *T. tesselatus* was not well-supported, (2) clarify the taxonomy and circumscription of *T. teuscheri*, and (3) lump *T. isabelae* with *T. teuscheri*. In addition, we provide an updated description of *T. teuscheri* and information on its distribution, ecology, molecular affinities, and conservation status.

## Materials and methods

### Bibliographic, herbarium and photographic revision

To clarify the taxonomy of the orchid species here studied, we conducted an extensive revision of herbarium specimens, original species descriptions and illustrations (e.g. Garay 1958, Dodson and Bennett 1989, Dodson and Dodson 1989, Dodson 2002, 2004). We also examined material deposited at several herbaria such as: AMES, K, MO, QCA, QCNE, and SEL. Online databases were consulted to locate additional specimens in Tropicos.org (https://tropicos.org), JSTOR Global Plant database (https://plants.jstor.org/), iDigBio (https://www.idigbio.org/). Specimens were examined physically at Ecuadorian (QCNE, QCA) and British (K) herbaria (acronyms following Thiers 2026), and digitally for specimens deposited elsewhere. Original protologues were analyzed in detail to compare diagnostic morphological characters. In addition, we searched for photographs to cross-reference morphological features and assess potential intraspecific variation. The photographs were sourced from orchid enthusiasts in Ecuador, the Flickr image repository and the citizen science platform iNaturalist (https://www.inaturalist.org/). Photographs of specimens used in this study were downloaded from the Digital Collection of the Harvard University Herbaria (https://www.huh.harvard.edu/), Tropicos.org of the Missouri Botanical Garden (2025) and The Kew Data Portal (2025).

### Field surveys, morphological and ecological documentation

To confirm the species identity and evaluate morphological variability in natural populations, we conducted fieldwork in the type localities of *Telipogon isabelae, T. tesselatus*, *T. teuscheri* and similar species such as *T. tamboensis* Dodson & Hirtz and *T. thomasii* Dodson & R.Escobar, along with additional locations in nearby areas with similar ecosystems where these species were expected to occur. *Telipogon* plants were collected as voucher specimens. Flowers were dissected for morphological analysis. While vegetative and some floral material were pressed and dried according to standard herbarium procedures, some flowers were preserved in Copenhagen solution (70% ethanol, 29% water and 1% glycerol). Additionally, leaf samples were dried in silica gel for posterior DNA extraction and genetic analyses. All voucher specimens were deposited in the National Herbarium of Ecuador (QCNE). Photographs were taken in situ to document habitat, plant habit, and flower morphology, while detailed photographs with scale references were taken ex situ. Morphological measurements of vegetative and floral structures were obtained using ImageJ 1.53m (Rasband 2025), based on calibrated photographs of fresh material in extended position. During fieldwork, we observed the following material: *T. tamboensis*: 5 flowers/4 plants/1 population (near Zhud, Cañar Province, Aug/2022 and Jun/2023); *T. tesselatus*: 5 flowers/5 plants/5 populations (near Saraguro, Oct/2022 and Sep/2024, near Loja, Feb/2024 in Loja Province, east of Cuenca, Azuay Province, Dec/2022, 2023); *T. teuscheri*: 10 flowers, 7 plants, 2 populations (near Portete, Dec/2021, Aug/2022, Jun/2023, Near Mazán, Azuay Province, Sep/2023), and *T. thomasii*: 5 flowers, 3 plants, 2 populations (near Molleturo, Azuay Province, Aug/2022). Photographs were taken using a Nikon D5100 DSLR camera equipped with an AF-S DX Micro Nikkor 40 mm f/2.8G lens. Photographs were then used to prepare figures, which were edited using Adobe Photoshop® 2019. The ecosystem types in which the species occur were identified and classified using the official Ecuadorian Ecosystem Classification System (Ministerio del Ambiente 2013; MAATE 2025). A distribution map was prepared using QGIS (version 3.40.11-Bratislava; https://qgis.org), with basemap from OpenTopoMap (Kartendaten: © OpenStreetMap-Mitwirkende, SRTM | Kartendarstellung: © OpenTopoMap | CC-BY-SA license). Spatial layers were projected under WGS 84 coordinate system (EPSG:4326). The extent of occurrence (EOO) and the area of occupancy (AOO–based on a cell size of 4 km^2^) were calculated using the GeoCAT tool (https://geocat.iucnredlist.org/, Bachman et al. 2011), to prepare a preliminary conservation assessment following IUCN (2024) criteria. Botanical terminology used in morphological descriptions follows Beentje (2012).

### Phylogenetic analysis using nuclear and plastid markers

Molecular analyses were conducted to infer the phylogenetic relationships between the studied species. Genomic DNA was extracted from silica-dried leaf material using a rapid extraction protocol of Kasajima et al. (2004) in the laboratories of the Universidad de Las Américas (UDLA), Quito, Ecuador. Two DNA markers were amplified: the nuclear ribosomal internal transcribed spacer (nrITS; hereafter ITS) and the plastid *maturase K* (*matK*) gene. PCR reactions were carried out in a final volume of 25 μL, including 7.5 μL GoTaq Green Master Mix 2× (Promega), 3 μL of template DNA, 0.75 μM of each primer, and ultrapure water. Primer sequences and PCR cycling conditions followed Iturralde et al. (2023). PCR products were purified and sequenced bidirectionally using Sanger sequencing (on an ABI 3500xL Genetic Analyzer, Applied Biosystems).

New sequences obtained from this study were deposited in GenBank (see Suppl. material 1). Comparative sequences of *Telipogon* species from Central and South America were retrieved from GenBank (https://www.ncbi.nlm.nih.gov/genbank/) to construct a broader phylogenetic framework. In total, 24 *Telipogon* species were included, along with *Hofmeisterella eumicroscopica* (Rchb.f.) Rchb.f. and *Trichoceros antennifer* (Bonpl.) Kunth, members of the *Telipogon* alliance (Williams et al. 2005; Martel et al. 2020). *Fernandezia sanguinea* (Lindl.) Garay & Dunst. (Orchidaceae: Oncidiinae) was selected as the outgroup (Suppl. material 1).

Sequence alignments, trimming, concatenation and phylogenetic analyses were performed in Geneious Prime 2022.1 (https://www.geneious.com/). Sequence alignments were generated using the Geneious alignment algorithm (default settings). The resulting alignments were inspected visually, and terminal regions containing more than 50% gaps across sequences were trimmed. The dataset was relatively small, no ambiguously aligned regions were detected, and automated trimming was unnecessary.

Phylogenetic trees were reconstructed using Bayesian Inference (BI) and Maximum Likelihood (ML). The best substitution model was estimated with MEGA 11.0.13 (Tamura et al. 2021). BI analyses were implemented with the MrBayes 3.2.6 plugin (Huelsenbeck and Ronquist 2001), and ML analyses using RAxML v8.2.11 plugin (Stamatakis 2014). Both analyses were conducted on independent ITS and *matK* alignments, and a partitioned concatenated dataset (ITS: 1–636; *matK*: 637–1763).

For BI, each marker was assigned its best‑fit model: HKY+G+I for ITS and HKY+I for *matK*. For the concatenated matrix, two partitions were defined according to gene boundaries and assigned the same models. Two independent MCMC runs of four chains were executed for 10 million generations, sampling every 10,000 generations, with a 25% burn‑in. Convergence was assessed by monitoring average standard deviation of split frequencies < 0.02, PSRF values ~1.0, and ESS > 200. A 50% majority‑rule consensus tree was generated, with posterior probabilities used as nodal support.

For ML, independent analyses of ITS, *matK* and the partitioned concatenated datasets were conducted under the GTR+G+I model. We used the rapid bootstrap algorithm with 1000 replicates. Bootstrap values were mapped onto the best‑scoring ML topology and used as nodal support.

## Results and discussion

### Untangling the identity of *Telipogon isabelae*

**Figure 1.**
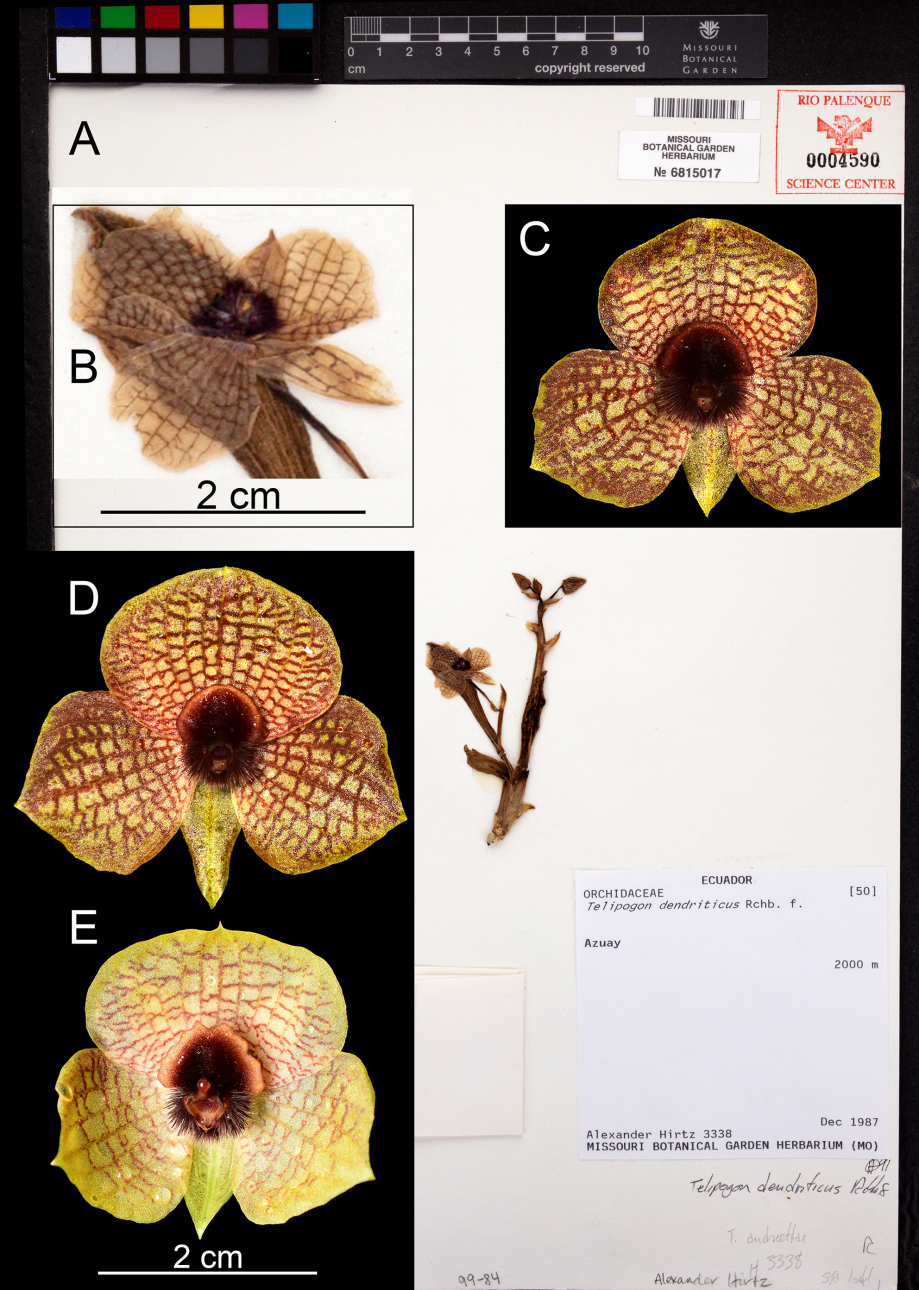
*Telipogon teuscheri*. **A**. Holotype of *T. isabelae A. Hirtz 3338* (MO! – ex RPSC) herein synonymized with *T. teuscheri*; **B**. Close-up of the preserved flower; **C**. Flower from specimen found near Mazán (*F. Sánchez & G.A. Iturralde GI-2309-1907*QCNE!); **D**. Flower from specimen found near Portete (*Gabriel A. Iturralde GI-2305-1083*QCNE!); **E**. Another flower from same locality in Portete (not collected). Images from Tropicos.org, Missouri Botanical Garden (**A, B**) [accessed 30 April 2025].

The protologue of *Telipogon isabelae* (Dodson 2004) designates *Hirtz 3338* (ex RPSC – MO!) as the holotype (Fig. 1), collected near Santa Isabel, Azuay Province. However, the species was illustrated with photographic plate #2420 (pp. 1044; Dodson 2004), which depicts flowers belonging to *T. thomasii* Dodson & R.Escobar (also available at https://www.tropicos.org/specimen/103339812). Furthermore, the protologue describes the corolla as covered with a red-brown haze, the callus as cordiform, and the dorsal column’s surface covered in dense, short red-brown setae (pp. 1183; Dodson 2004). These characteristics, together with the plate #2420, match the description and morphological characteristics of *T. thomasii* (see plate 597; Dodson and Dodson 1989). The holotype of *T. isabelae*, however, shows a flower with strong reticulation and any corolla haze, and a column with 3 tufts of long dark red setae (Fig. 1).

**Figure 2.**
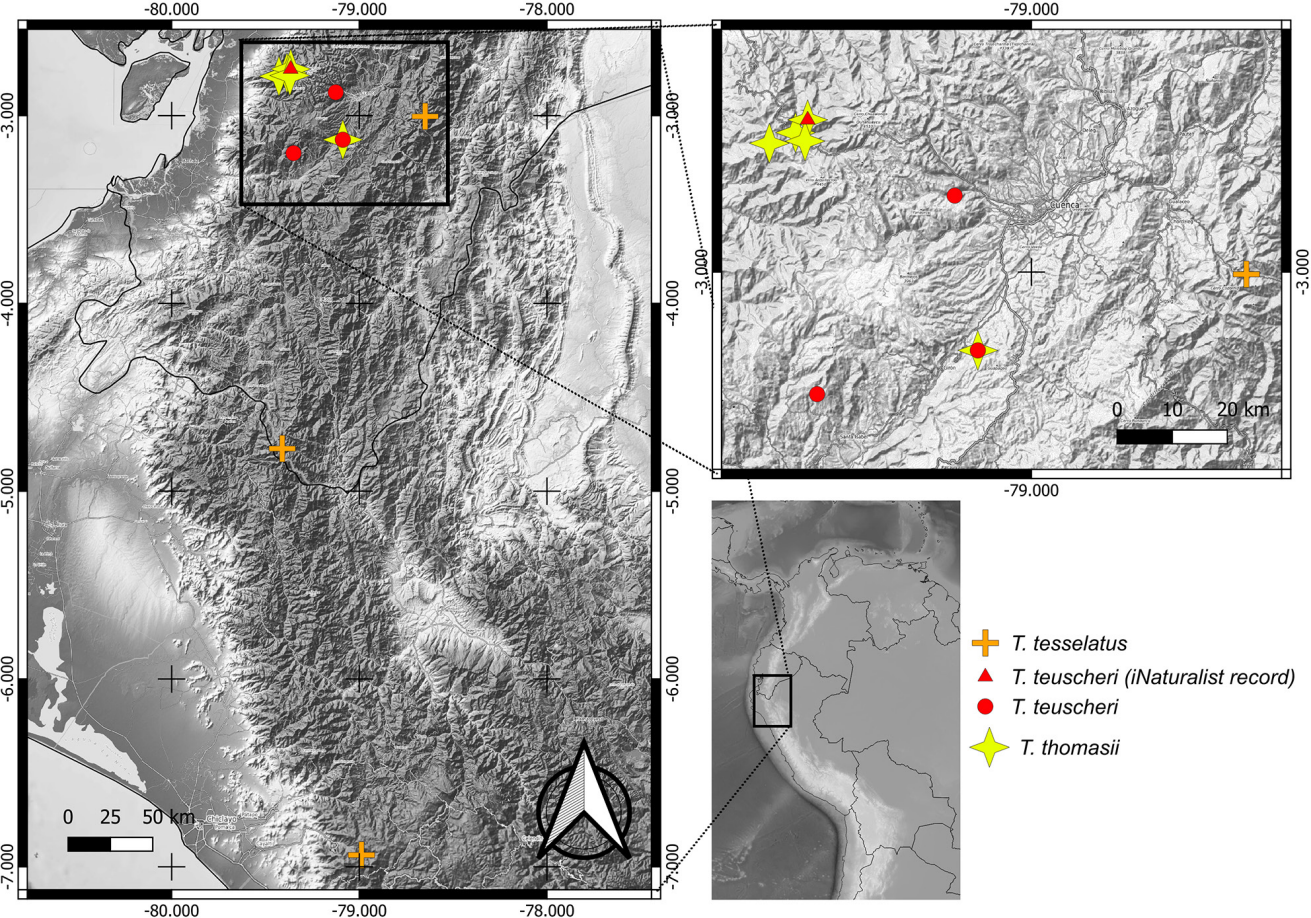
Distribution of *Telipogon teuscheri* and morphologically similar species in the northern Andes (Ecuador, Peru).

Our recent fieldwork in Azuay Province (localities near Portete, close to Santa Isabel, and near Cuenca) supports our interpretation. Plants from these populations match the morphology of the holotype of *T. isabelae* (Figs 1, 2). Furthermore, we found individuals of *T. thomasii* growing sympatrically with plants resembling the holotype of *T. isabelae* near Portete, even on the same branches, which may explain the discrepancy between the species description, illustration and the holotype.

**Figure 3.**
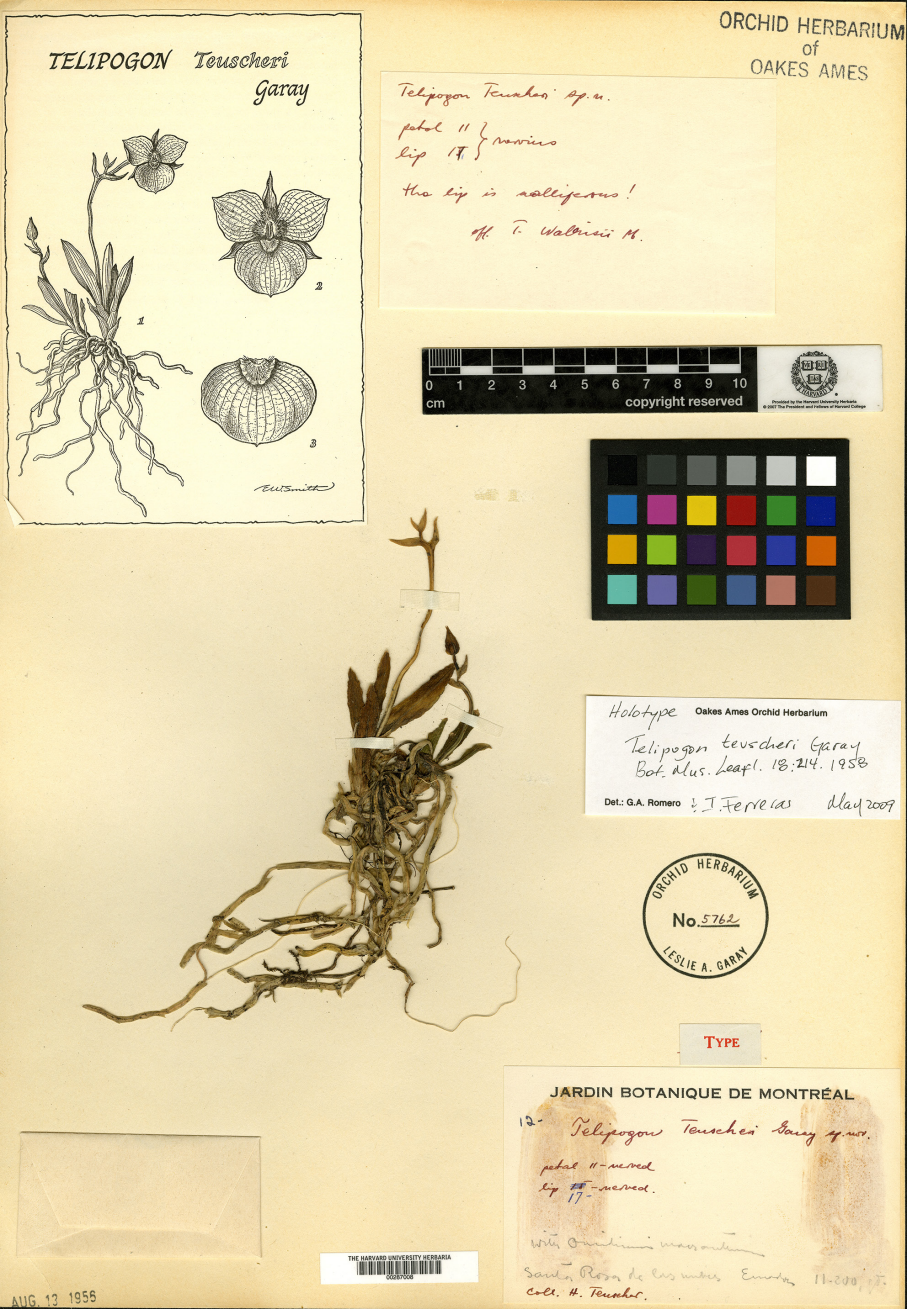
Holotype of *Telipogon teuscheri* (*H. Teuscher 12* – AMES! 00287008). Image from The Orchid Herbarium of Oakes Ames of Harvard University.

After the revision of the reticulate-flowered species of *Telipogon* from Ecuador, the type specimen (*H. Teuscher 12* – AMES!, Fig. 3) and protologue of the long-forgotten *T. teuscheri* (Garay 1958) were found to correspond to the morphological traits seen in the type of *T. isabelae* as well as those observed in individuals found in the type and nearby localities. In the protologue of *T. teuscheri*, the flowers were described as medium-sized for the genus, and the lip was described as having a deltoid callus with hirsute and rigid hairs, and most importantly, a detailed illustration was included alongside the description of the species (Fig. 3), all of which unequivocally match those of the type specimen of *T. isabelae* and specimens near the type locality. Although the type locality of *T. teuscheri* was indicated as Santa Rosa de las Nubes, no locality with this name currently exists in Ecuador. Nevertheless, the town of Octavio Cordero Palacios, near Cuenca, was previously known as Santa Rosa. Moreover, historical records show that Heinrich Teuscher (1891–1984), the collector of the type, explored montane areas near Cuenca and along the Cuenca–Guayaquil Road, where he collected other plant specimens around the same date (e.g., *H. Teuscher s.n*., USF [26887], [29288], [29114]; *H. Teuscher s.n*., AMES [00271997]). Therefore, we hypothesize that the type of *T. teuscheri* may have come from the mountains around Octavio Cordero Palacios, Azuay Province.

### Reassessment of *Telipogon teuscheri* based on morphological and genetic evidence

**Figure 4.**
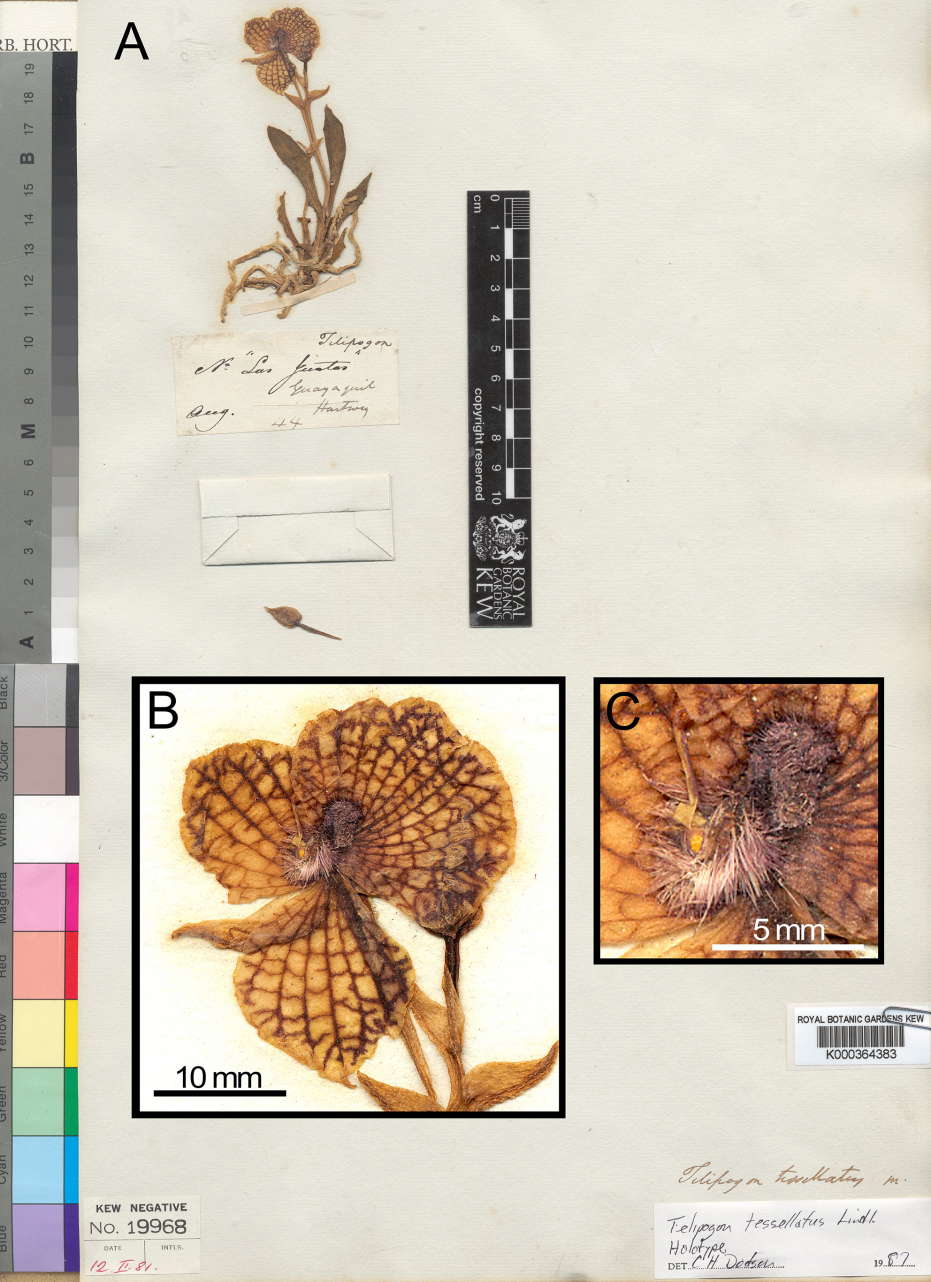
*Telipogon tesselatus*. **A**. Holotype (*Hartweg 44* – K! - K000364383; http://specimens.kew.org/herbarium/K000364383); **B**. Close-up of the preserved flower. Reproduced under the terms of the Creative Commons Attribution License [CC BY 4.0] (http://creativecommons.org/licenses/by/4.0). © The Board of Trustees of the Royal Botanic Gardens, Kew.

**Figure 5.**
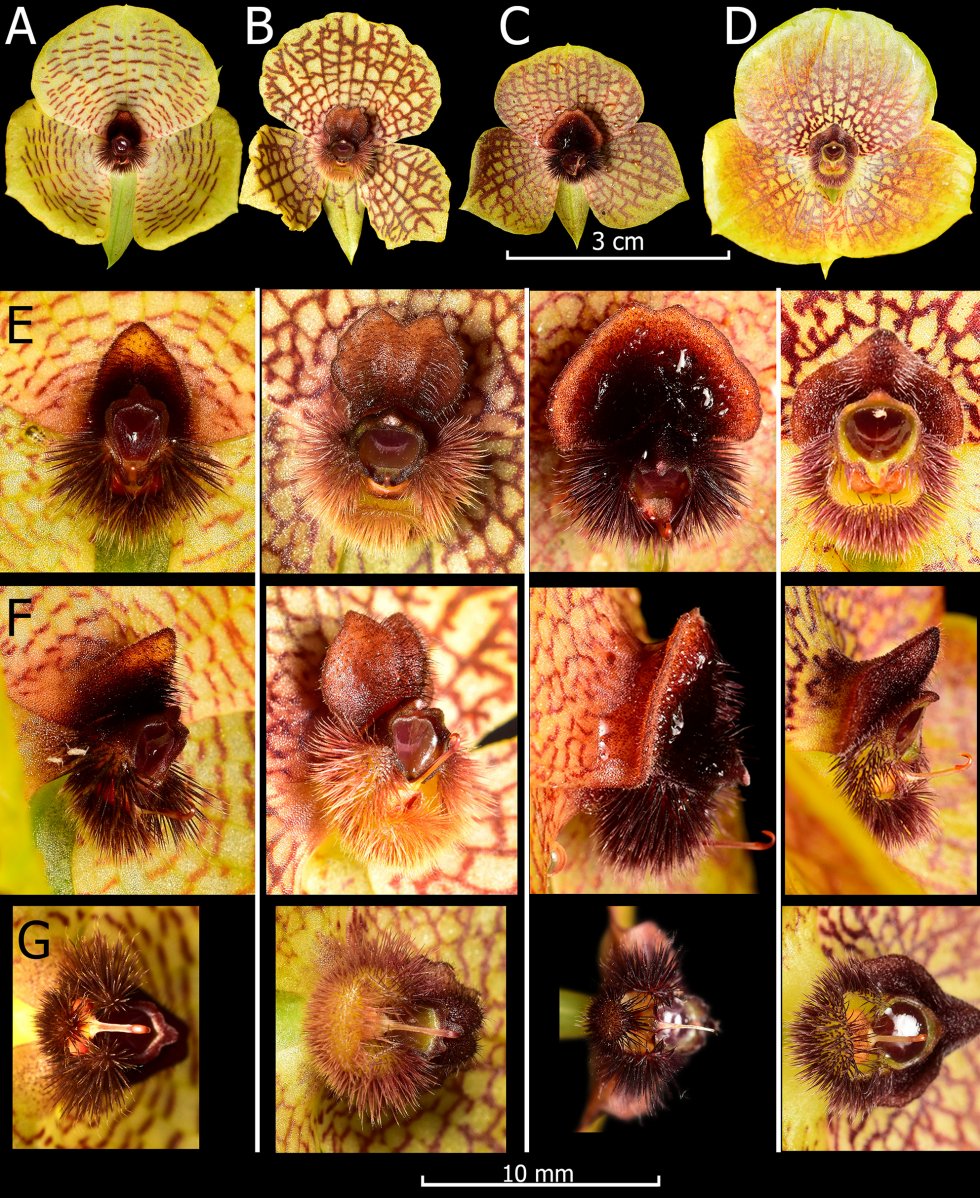
*Telipogon* species compared in this study. Flowers. **A**. *T. tamboensis* from specimen found near Zhud-Cañar (*Gabriel A. Iturralde GI-2305-0939*QCNE!); **B**. *T. tesselatus* from a specimen found near Saraguro-Loja (Photo # GI-2409-6249); **C**. *T. teuscheri* from specimen found near Portete-Azuay (Photo # GI-2306-1145); **D**. *T. thomasii* from a specimen found near Molleturo-Azuay (Photo # GI-2208-4745). Close-up of column and lip callus; **E**. Frontal view; **F**. Lateral view; **G**. Dorsal view. Photos by G. Iturralde.

**Figure 6.**
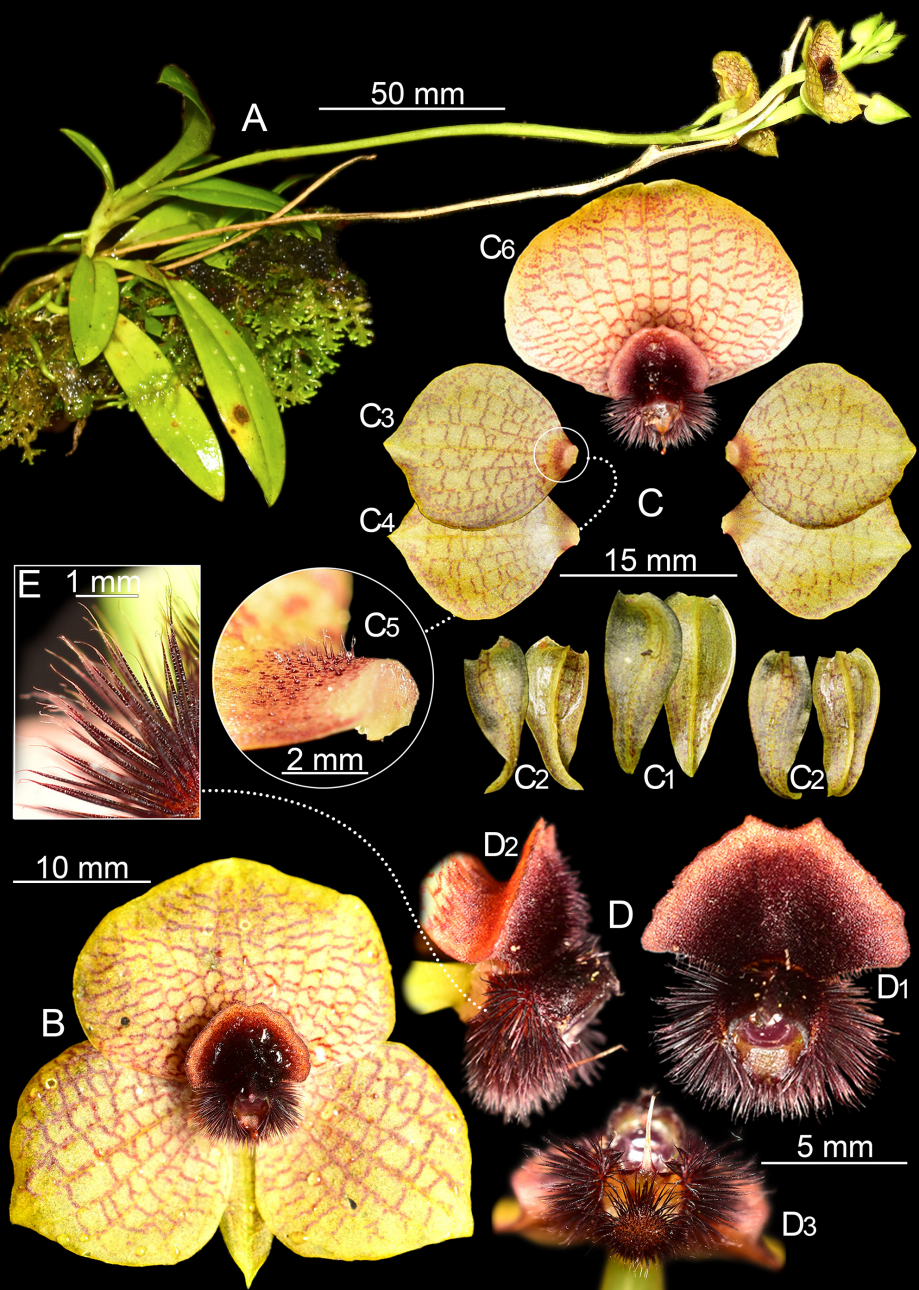
*Telipogon teuscheri* from near Portete-Azuay. **A**. Habit; **B**. Flower; **C**. Dissected perianth: **C1**. Dorsal sepal adaxial side (left), abaxial side (right); **C2**. Lateral sepals adaxial side (left), abaxial side (right); **C3**. Petals adaxial side; **C4**. Petals abaxial side; **C5**. Close-up of petal base; **C6**. Lip and column; **D**. Column and lip callus: **D1**. frontal view; **D2**. Lateral view; **D3**. Dorsal view; **E**. Close-up of column setae. Prepared by G.A. Iturralde based on *G.A. Iturralde GI-2208-5095* (QCNE!) (**A, D, E**) and *GI-2305-1083* (QCNE!) (**B, C**).

*Telipogon teuscheri* was described by Garay (1958) but was later reduced to the synonymy of *T. tesselatus* by Dodson (in Dodson and Bennett 1989) and has been accepted as such by recent taxonomic accounts and online databases (e.g. Dodson 2004; Szlachetko et al. 2022; Missouri Botanical Garden 2025; POWO 2025). However, the comparison of the types and protologues of these two species names does not support the synonymy. Although the holotype of *T. teuscheri* (*H. Teuscher 12* – AMES 00287008!, Fig. 3) consists of a flowerless plant, the species protologue includes a thorough description and an illustration showing a plant identical to that of the type specimen and a detailed drawing of a flower. Conversely, the protologue of *T. tesselatus* lacks diagnostic details, but the type specimen (*K.T. Hartweg 44* - K!, Fig. 4) features a specimen with a well-preserved flower in which the key characteristics, including the callus and column, can be observed. Although both species have similar-sized and reticulate flowers, two diagnostic characters clearly distinguish them: (1) The callus in *T. teuscheri* is lunate-deltoid, dark purple at the center with lighter margins, villose centrally and velvety toward the flattened edges, whereas it is subquadrate and bifid with a central longitudinal sulcus in *T. tesselatus*. (2) The column in *T. teuscheri* is dark purple and bears three distinct tufts of dark setae, none pointing beyond a perpendicular line in the reference to the axis of the callus (Figs 1, 3–6), whereas the column in *T. tesselatus* is yellow, the apical lobes are swollen, uniformly and profusely covered by red-yellow setae and some of which point out towards the direction of the callus (Fig. 5). These differences are consistent across examined specimens from the species type locations and nearby areas. Thus, specimens examined and identified as *T. teuscheri* seem to be restricted to the southern inter-Andean valleys of Ecuador, whereas *T. tesselatus* seems to be restricted to the southern Ecuador and northern Peru up to the Amotape-Huancabamba zone (Fig. 2). All this evidence supports that *T. teuscheri* is morphologically and geographically distinct from *T. tesselatus* (Fig. 5).

**Figure 7.**
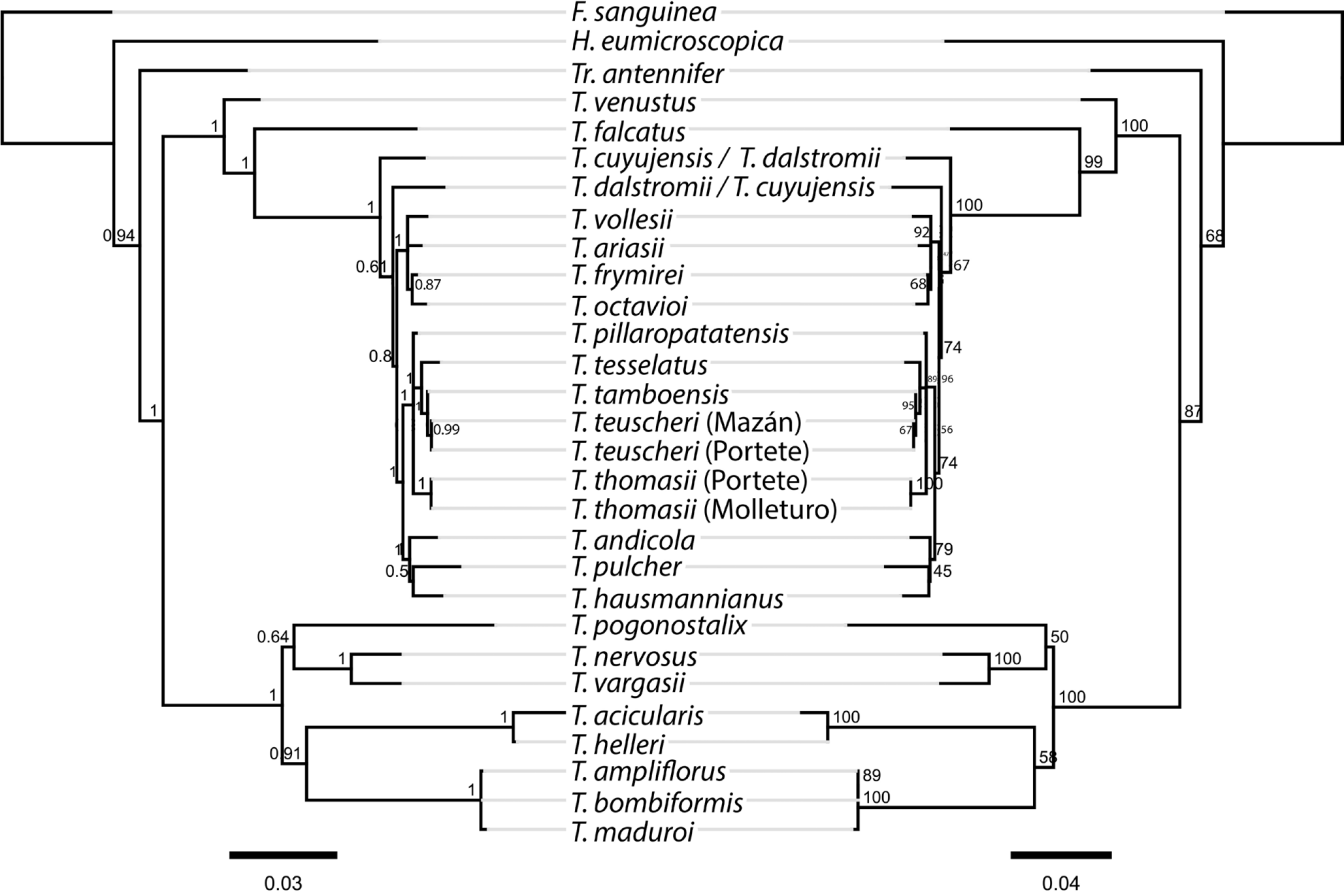
Reconstructed phylogenetic trees of concatenated markers nrITS and *matK*, including the position of *Telipogon teuscheri* with its relationships. Tree on the left based on Bayesian inference (BI) analysis (numbers in nodes represent posterior probability). Tree on the right based on Maximum Likelihood (ML) analysis (numbers in nodes represent bootstrap percentages). Scale bars represent the mean number of nucleotide substitutions per site.

Phylogenetic trees inferred from the ITS and *matK* datasets separately showed topologies largely congruent with the concatenated dataset, especially with respect to the placement and relationships of individuals assigned as *T. teuscheri* and *T. tesselatus*. Given the high consistency among the analyses, the increased resolution provided by the combined dataset, and to avoid redundancy, we present and discuss only the phylogenetic tree based on the concatenated matrix (Fig. 7).

Bayesian Inference (BI) and Maximum Likelihood (ML) analyses of the concatenated dataset yielded largely congruent topologies, recovering *Telipogon* as a monophyletic group with strong support (posterior probability [PP] = 1; bootstrap support [BS] = 87). Within the genus, several clades were well-supported, including a strongly supported clade containing *T. teuscheri* and *T. tesselatus*.

Accessions of *T. teuscheri* from two localities (Portete and Mazán) clustered together in a well-supported subclade (PP = 0.99; BS = 67). Although *T. teuscheri* and *T. tesselatus* were placed in the same clade, *T. teuscheri* was retrieved as more closely related to *T. tamboensis* (PP = 1; BS = 95, Figs 4, 5) and these two are sister to *T. tesselatus* (PP = 1; BS = 89). *Telipogon thomasii*, whose accessions came from Molleturo and Portete, also formed a well-supported subclade (PP = 1; BS = 100), which is distantly related to *T. teuscheri*. In the phylogenetic tree, *T. teuscheri* and *T. tesselatus* form distinct lineages, with *T. tamboensis* positioned between them, supporting their recognition as genetically distinct clades in our sampled markers.

### Taxonomic significance

The combined morphological and genetic evidence presented support the reinstatement of *Telipogon teuscheri* as a species distinct from *T. tesselatus*. The reevaluation of type material, together with field observations and phylogenetic analyses, further demonstrates that the name *T. isabelae* is not a separate taxon. Accordingly, *Telipogon teuscheri* is reinstated as a distinct species, and *T. isabelae* is placed as its synonym.

### Taxonomic treatment: circumscription of *Telipogon teuscheri*

#### Telipogon teuscheri

Taxon classificationPlantaeAsparagalesOrchidaceae

Garay, Bot. Mus. Leafl. 18: 214, tab. 43. 1958.

87270A19-3CCB-5E41-8631-66D88079BE2C

Figs 1, 6

*= Telipogon isabelae* Dodson & Hirtz, Native Ecuadorian Orchids 5: 1183. 2004. Type: Ecuador, Azuay: *A. Hirtz 3338* (holotype: MO! – ex RPSC). syn. nov.

##### Type material.

**Ecuador • Azuay**, Santa Rosa de las Nubes [Octavio Cordero Palacios], 13 Aug 1956: *H. Teuscher 12* (holotype: AMES!).

##### Description.

***Plant*** up to 24 cm long (including the inflorescence), epiphytic, caespitose, each new shoot produced appressed to and above the previous one. ***Roots*** 1.0–1.3 mm in diameter, cylindrical, basal. ***Stem*** on mature plants forming pseudobulb-like structures, up to 10 × 2.5 mm, arising from a rhizome and with a single leaf at the apex. ***Leaves*** up to 4 per stem, distichous, subcoriaceous, slightly carinate abaxially, articulated to decurrent, conduplicate leaf sheaths completely covering the stem; the basal leaves smaller than the upper leaves; ***blade*** 20–110 × 5.9–19 mm, conduplicate, narrowly elliptic-obovate, acute, slightly apiculate. ***Inflorescence*** raceme up to 210 mm long, lateral or apical; ***peduncle*** green, simple, narrow at the base and gradually broadening towards the rachis, becoming tetragonal; ***rachis*** up to 40 mm long, ancipitous, producing apically 2–7 spirally arranged flowers, opening in succession, 1(–2) simultaneously; ***floral bracts*** 6.5–11.0 mm long, green, membranaceous, conduplicate, triangular, boat-shaped, ovate when extended, acute, carinate abaxially, sometimes with a small, thin bracteole at the opposite side. ***Ovary*** 22.6–38.0 mm long, triquetrous, winged; ***pedicel*** 1–3 mm long, terete. ***Flowers*** non-resupinate; ***sepals*** yellow with dark red-purple irregular lines, ovate, acute, concave, carinate abaxially, obscurely 3-veined; ***dorsal sepal*** 12.5–18.0 × 4.6–7.9 mm; ***lateral sepals*** 13.2–15.8 × 4.7–6.1 mm, oblique. ***Petals*** 14.5–20.1 × 12.8–19.8 mm, yellow with dark yellow longitudinal veins suffused with dark red-brown reticulations, broadly elliptic-rhomboid, acute at the apex, margins slightly undulate, (8–)9–11-veined; glabrous, with minutely ciliate base. ***Lip*** 15.2–19.3 × 20.5–27.1 mm, with the same color pattern of the petals, transversally obovate, obtuse and shortly apiculate at the apex, the margins slightly undulate; 15(–19)-veined, glabrous; ***lip callus*** 5.4–6.7 × 6.2–10.1 mm, dark purple at the center with yellow-reddish margins, protruding ventrally from the base of the lip, free from the blade for ca. 3 mm, lunate-deltoid, convex, profusely villose centrally, turning flat and velvety towards the irregular margins that form a skirt, the apex varying from broadly obtuse, retuse to emarginate. ***Column*** ca. 4.5 mm long up to ca. 4.0 mm in diameter (without including setae), dark purple, sessile, slightly velvety, dorsally trilobed, lateral lobes slightly swollen, central lobe dactyliform, curved toward the clinandrium and covering half of the anther cap; each lobe bearing a dense tuft of dark purple, simple, acicular setae with pale whitish tips; central tuft shorter 1.8–2.1 mm long, lateral tufts longer 2.4–3.3 mm long; ***clinandrium*** concave with rounded edges. ***Stigma*** ca. 2.2 mm in diameter, dark purple, apical, circular, covered by a translucent, sticky secretion, with thin, dark purple, slightly sinuate margins, protruding ca. 1 mm towards the lip. ***Anther cap*** ca. 1.8 × 2.4 mm, hidden under the clinandrium cavity, dorsal, cordiform, yellow-orange. ***Pollinarium*** not observed. ***Fruit*** a carinate capsule.

##### Additional examined material.

**Ecuador: Azuay** • El Bosque de Ingapucará, cerca de Huasipamba (Sta. Isabel), 2720–3080 m, 9 Aug 1990, *R. Macklin 50* (QCA!); • Plants grown in the greenhouses of Father Angel Andreetta in Paute, 22 km east of Cuenca on the Río Paute, Dec 1988, *A. Hirtz 3339* (MO – ex RPSC photo!); • southeast of Portete, 3000 m, 6 May 2022, *G.A. Iturralde GI-2305-1117* (QCNE!); • Ibid. loc., 1 Jun 2022, *G.A. Iturralde GI-2208-5095* (QCNE!) • Ibid. loc., 13 Jun 2023, *G.A. Iturralde*, *GI-2305-1083* (QCNE!); • Mazán, vía a El Cajas, 3100 m, 13 Sep 2023, *F. Sánchez & G.A. Iturralde GI-2309-1907* (QCNE!).

##### iNaturalist records.

Ecuador: Azuay, near Cuenca, Aug 2023, *fkarste*https://www.inaturalist.org/observations/176645191 (Suppl. material 2: fig S1); near San Felipe de Molleturo, 30 Jul 2025, *fkarste*https://www.inaturalist.org/observations/302520547 (Suppl. material 2: fig S2) and https://www.inaturalist.org/observations/302520681 (Suppl. material 2: fig S3).

##### Taxonomic notes.

*Telipogon teuscheri* is characterized by the yellowish flowers with a prominent reddish reticulate pattern extending to the margins of the corolla, the petals and lip not exceeding 20.1 mm in length, the large lunate-deltoid callus reaching up to 1/3 of the lip, villose centrally, velvety and flat towards the margins, forming a skirt whose apex ranges from broadly obtuse, retuse, to emarginate. In summary, the reticulate pattern and the lunate-deltoid and villose-velvety callus of the lip with a lighter colored margin allow *T. teuscheri* to be easily differentiated from any other currently known *Telipogon* species (Figs 1, 5C, 6).

As stated above, *Telipogon teuscheri* has flowers similar in size and reticulation to those of *T. tesselatus* (Fig. 5B) but differs in the broadly elliptic-rhomboid petals (vs. suborbicular to ovate-suborbicular), the transversely obovate lip (vs. transversely elliptic), the lunate-deltoid and villose-velvety callus of the lip (vs. the subquadrate, bifid callus with a central longitudinal sulcus), and the dark brown-purple column with three clearly distinct tufts of dark purple setae (vs. yellow column uniformly and profusely covered with red-yellow setae).

*Telipogon teuscheri* is also similar to *T. thomasii* and *T. tamboensis*. A comparison of floral traits is summarized in Table 1 and illustrated in Fig. 5.

**Table 1.** Summary of floral characteristics of *Telipogon teuscheri* and morphological similar *Telipogon* species.

	*T. tamboensis*	*T. tesselatus*	*T. teuscheri*	*T. thomasii*
Corolla	Pale cream-yellow with longitudinal dark yellow veins and conspicuous transverse, short reddish-brown lines arranged to form circles in the corolla	Yellow with red-brown reticulate lines	Yellow with red-brown reticulate lines	Tan-yellow with red-brown reticulate lines
Petals	15–21 × 12–23.5 mm, elliptic-ovate, apex acuminate	15–27.1 × 12–18.5 mm, suborbicular to ovate-suborbicular, apex shortly acuminate	14.5–20.1 × 12.8–19.8 mm, broadly elliptic-rhomboid, apex acute	20–25.8 × 20–27.5 mm, broadly elliptic, apex obtuse and apiculate
# petal veins	(8–)9–11	(6–)7–8	(8–)9–11	(8–)10–13
Lip	18–19.8 × 13–26.5 mm, broadly elliptical, apex obtuse, apiculate	14–25.8 × 21–32.0 mm, transversely elliptic, apex obtuse to shortly acuminate	15.2–19.3 × 20.5–27.1 mm, transversally obovate, apex obtuse and shortly apiculate	18.4–24.6 × 23.9–31.7 mm, broadly ovate-elliptic, apex obtuse, apiculate
# lip veins	(11–)13–15	(14–)15–17	15(–19)	(14–)15–18
Lip callus	Red-brown, lighter toward the apex, narrowly cordiform, villose-hirsute	Reddish-purple, subquadrate, convex, hispid, with a central longitudinal sulcus, margins thin, skirt-like, apex bifid	Dark purple, lighter at margins, lunate-deltoid, convex, turning flat, skirt-like on margins, villose centrally, velvety on margins, apex broadly obtuse, retuse, to emarginate	Dark red to brown, subcordiform, hirsute, with a longitudinal central ridge, sub-trilobed
Column	Red-brown, dorsally trilobed, lateral lobes slightly swollen, central lobe dactyliform and curved to the clinandrium, with 3 tufts of evenly long setae, one per lobe	Yellow, dorsally trilobed, lobes swollen, upper lobe wide and curved to the clinandrium, lobes densely and evenly setose	Dark brown-purple, dorsally trilobed, lateral lobes slightly swollen, central lobe dactyliform and curved to the clinandrium, with 3 tufts of setae, one per lobe, central tuft shorter than lateral	Yellow, dorsally trilobed, central lobe wide, slightly swollen and curved to the clinandrium, lobes evenly short setose
Column setae	Red-brown, acicular, ca. 3 mm long	Red-yellow, acicular, ca. 3 mm long	Dark purple, acicular, central 1.8–2.1 mm long, lateral 2.4–3.3 mm long	Purple, acicular, ca. 2 mm long

##### Distribution, habitat, and ecology.

Specimens of *T. teuscheri* have been recorded in three localities restricted to the province of Azuay, in the Interandean Valleys of southern Ecuador (Fig. 2). One locality is north of Santa Isabel, the second is southeast of Portete, and the third is west of Cuenca. Additionally, there is an unexpected iNaturalist record of *T. teuscheri* near Molleturo, at approximately 2400 m a.s.l., 40 km northwest of Cuenca, which would extend its longitudinal range toward the western foothills of Azuay as well as its altitudinal range. Further expeditions to this area are needed to collect material and confirm this finding.

The elevation of 2000 m indicated in the holotype of *T. isabelae* (Fig. 1) is somewhat unusual and likely erroneous, as habitats at that elevation near Santa Isabel correspond to warm semi-deciduous forest and shrublands (Ministerio del Ambiente 2013). Acaulescent *Telipogon* species with angled inflorescences and medium-sized to large flowers usually inhabit higher elevations, from ca. 2400 m to 3300 m a.s.l.

Therefore, based on species records apart from the holotype, we suggest that the most probable elevational range of the species is 2700–3100 m a.s.l. The corresponding ecosystems are the montane and high montane evergreen forests (codes BsMn02, BsMn03 and BsAn03). These forests are characterized by mean annual temperatures ranging from 10.1 to 16.2 °C and canopies between 15 and 25 m tall. At increasing elevations, tree trunks become thick and twisted, with many individuals branching at ground level or developing adventitious roots. The understory is notably dense and enriched with herbaceous plants, epiphytes, and bryophytes that extensively cover the forest floor (Ministerio del Ambiente 2013). The largest population of *T. teuscheri* observed occupied approximately 400 m^2^, within a patch of very dense forest, located in a humid ravine. Within the understory, at least 50 individuals were counted. Most of the plants were young plants in their first or second flowering, and only some were large, mature plants. Many plants were anchored on thin branches located less than two meters above ground and a few on thicker branches located up to four meters above ground. In the same forest patch, we also observed individuals of *T. thomasii*, and other orchids such as *Elleanthus aurantiacus* (Lindl.) Rchb.f., *Fernandezia debedoutii* (P.Ortiz) M.W.Chase, *F. maculata* Garay & Dunst., *F. hispidula* (Rchb.f.) M.W.Chase, and *Malaxis histionantha* (Otto) Garay & Dunst.

##### Phenology and flower variation.

Herbarium specimens of *T. teuscheri* with flowers were collected in August and December. We collected plants flowering from May to September, while in December, plants from the Portete locality were without flowers. The showy flowers are probably pollinated by male tachinid flies as previously described for other showy *Telipogon* species (Martel et al. 2016, 2019), which use a pollination mechanism based on sexual deceit. *Telipogon teuscheri* presents a remarkable flower variation in size, the color intensity of the reticulations on the petals, and the lip callus shape. Petals and lip length ranges from 14.5–20.1 mm and 15.2–19.3 mm, respectively. The petals can be almost completely yellow with a barely perceptible reticulation, but they can also be very stained with thick dark-brown reticulations and a dark haze towards the edge of the corolla (Figs 1, 5). These variations have also been observed in *T. pillaropatatensis* Iturralde, Monteros & Baquero, where it is presumed that size is associated with plant age, and direct sunlight might be critical in inducing the production of the pigments responsible for the dark brown colorations of the corolla (Iturralde et al. 2023). On the other hand, calli vary from having a completely rounded border to an almost trapezoidal, sinuate-undulate border. Finally, the coloration of the callus margin can vary from purple to yellow, but it is always lighter than the center.

##### Preliminary conservation status.

The extent of occurrence (EOO) calculated for *Telipogon teuscheri* is 427.35 km^2^, and the area of occupancy (AOO) is 12 km^2^. The montane and high-montane evergreen forests where *T. teuscheri* occurs are highly fragmented due to anthropogenic activities, mainly livestock grazing. These activities have reduced continuous forest cover, resulting in numerous isolated patches. According to the ecosystem map of Azuay Province, the combined remaining area of these three ecosystems is approximately 1,040 km^2^ (~12.5% of the total area of the Azuay Province – Gobierno Provincial del Azuay 2022). Given the restricted AOO, the small and fragmented EOO, and ongoing habitat degradation, *T. teuscheri* meets the IUCN (2024) criteria for Endangered (EN) under categories B1ab(iii)+2ab(iii).

### Final remarks

Our results clarify a long-standing confusion in the taxonomy of *Telipogon* from southern Ecuador. The mismatch between the holotype of *T. isabelae* and its protologue likely resulted from Dodson inadvertently using photographs of *T. thomasii*, which grows sympatrically with *T. teuscheri*, for the description. At the same time, Dodson (2004) overlooked the similarity between *A. Hirtz 3338* and the type of *T. teuscheri*, perhaps influenced by his earlier synonymization with *T. tesselatus*.

By integrating different approaches, including morphological and phylogenetic analyses and field exploration, we propose that *T. teuscheri* should be regarded as a distinct species from *T. tesselatus*, and that *T. isabelae* should be considered a synonym of *T. teuscheri*. Given the narrow distribution of *T. teuscheri*, only known from the Azuay Province, and in fragmented montane forests subject to deforestation and habitat degradation, the species should be considered of conservation concern.

## Supplementary Material

XML Treatment for Telipogon teuscheri

## Additional information

### Use of AI

AI was used for English language editing. All text was carefully reviewed and approved by the authors.

### Author ORCIDs

Luis E. Baquero https://orcid.org/0000-0002-1444-5727

Carlos Martel https://orcid.org/0000-0001-9892-1999

Yanua Ledesma https://orcid.org/0000-0003-4139-3018

Mónica Salomé Guerrero-Freire https://orcid.org/0000-0001-8377-7816

Gabriel A. Iturralde https://orcid.org/0000-0003-2456-0929

### Data availability

All of the data that support the findings of this study are available in the main text or Supplementary Information.

## Supplementary materials

Supplementary material 1 GenBank accessions Data type docx Explanation note **table SS1**. GenBank accessions for the phylogenetic reconstruction. *Indicates sequenced vouchers for this study. https://binary.pensoft.net/file/1589128This dataset is made available under the Open Database License (http://opendatacommons.org/licenses/odbl/1.0/). The Open Database License (ODbL) is a license agreement intended to allow users to freely share, modify, and use this Dataset while maintaining this same freedom for others, provided that the original source and author(s) are credited. Luis E. Baquero, Carlos Martel, Yanua Ledesma, Mónica Salomé Guerrero-Freire, Gabriel A. Iturralde

Supplementary material 2 Supplementary figures Data type docx Explanation note **fig S1**. iNaturalist record of *Telipogon teuscheri* Garay – ECUADOR: Azuay, near Cuenca, Aug 2023, *fkarste*https://www.inaturalist.org/observations/176645191. **fig S2**. iNaturalist record of *Telipogon teuscheri* Garay – ECUADOR: Azuay, near San Felipe de Molleturo, 30 Jul 2025, *fkarste*https://www.inaturalist.org/observations/302520547. **fig S3**. iNaturalist record of *Telipogon teuscheri* Garay. – ECUADOR: Azuay, near San Felipe de Molleturo, 30 Jul 2025, *fkarste*https://www.inaturalist.org/observations/302520681. https://binary.pensoft.net/file/1589129This dataset is made available under the Open Database License (http://opendatacommons.org/licenses/odbl/1.0/). The Open Database License (ODbL) is a license agreement intended to allow users to freely share, modify, and use this Dataset while maintaining this same freedom for others, provided that the original source and author(s) are credited. Luis E. Baquero, Carlos Martel, Yanua Ledesma, Mónica Salomé Guerrero-Freire, Gabriel A. Iturralde
