# Supplementary material for: Morphological and genetic evidence support the reinstatement of the long-forgotten Telipogon teuscheri (Orchidaceae, Oncidiinae) from southwestern Ecuador
Source: PhytoKeys. 2026 Apr 9;273:1–20. doi: 10.3897/phytokeys.273.180600 (PMC13087665; doi:10.3897/phytokeys.273.180600)
Supplement: Supplementary material 1 — GenBank accessions [file phytokeys-273-001_article-180600__-s001.docx]

**Supplementary Table S1.** GenBank accessions for the phylogenetic reconstruction. *Indicates sequenced vouchers for this study.

| **Taxon** | **nrITS** | ***matK*** |
| --- | --- | --- |
| *Fernandezia sanguinea* (Lindl.) Garay & Dunst. | FJ565526 | FJ565009 |
| *Hofmeisterella eumicroscopica* (Rchb.f.) Rchb.f. | DQ315823 | AF350589 |
| *Telipogon acicularis* (Dressler) N.H.Williams & Dressler | DQ315837 | DQ315896 |
| *Telipogon ampliflorus* C.Schweinf. | DQ315850 | FJ564870 |
| *Telipogon andicola* Rchb.f. | DQ315851 | - |
| *Telipogon ariasii* Dodson & D.E.Benn. | DQ315852 | DQ315902 |
| *Telipogon bombiformis* Dressler | DQ315854 | FJ564866 |
| *Telipogon cuyujensis* Dodson & R.Escobar | OR689557 | OR689581 |
| *Telipogon dalstromii* Dodson | DQ315861 | DQ315906 |
| *Telipogon falcatus* Linden & Rchb.f. | DQ315862 | - |
| *Telipogon frymirei* Dodson | DQ315863 | - |
| *Telipogon hausmannianus* Rchb.f. | OR689558 | OR689580 |
| *Telipogon helleri* (L.O.Williams) N.H.Williams & Dressler | MF962882 | MF962888 |
| *Telipogon maduroi* Dressler | DQ315867 | FJ564867 |
| *Telipogon nervosus* (L.) Druce | DQ315870 | DQ315907 |
| *Telipogon octavioi* Dodson & R.Escobar | OR689559 | OR689579 |
| *Telipogon pillaropatatensis* Iturralde, Monteros & Baquero | OR689556 | OR689582 |
| *Telipogon pogonostalix* Rchb.f. | AF239392 | AF239488; |
| *Telipogon pulcher* Rchb.f. | DQ315875 | DQ315910 |
| **Telipogon tamboensis* Dodson & Hirtz | PX961057 (voucher GI-2305-0939) | PX964206 (voucher GI-2305-0939) |
| **Telipogon tesselatus* Lindl. | PX961058 (voucher GI-2211-7203) | PX964207 (voucher GI-2211-7203) |
| **Telipogon teuscheri* Garay (Mazán-Azuay) | PX961059 (voucher GI-2309-1907) | PX964208 (voucher GI-2309-1907) |
| **Telipogon teuscheri* Garay (Portete-Azuay) | PX961060 (voucher GI-2208-5095) | PX964209 (voucher GI-2208-5095) |
| **Telipogon thomasii* Dodson & R.Escobar (Portete-Azuay) | PX961061 (voucher GI-2305-1090) | PX964210 (voucher GI-2305-1090) |
| **Telipogon thomasii* Dodson & R.Escobar (Molleturo-Azuay) | PX961062 (voucher GI-2208-4737) | PX964211 (voucher GI-2208-4737) |
| *Telipogon vargasii* C.Schweinf. | DQ315880 | DQ315912 |
| *Telipogon venustus* Schltr. | FJ565183 | FJ564703 |
| *Telipogon vollesii* Dodson & R.Escobar | OR689560 | OR689578 |
| *Trichoceros antennifer* (Bonpl.) Kunth | DQ315883 | FJ564953 |
