# Supplementary material for: Morphological and genetic evidence support the reinstatement of the long-forgotten Telipogon teuscheri (Orchidaceae, Oncidiinae) from southwestern Ecuador
Source: PhytoKeys. 2026 Apr 9;273:1–20. doi: 10.3897/phytokeys.273.180600 (PMC13087665; doi:10.3897/phytokeys.273.180600)
Supplement: Supplementary material 2 — Supplementary figures [file phytokeys-273-001_article-180600__-s002.docx]

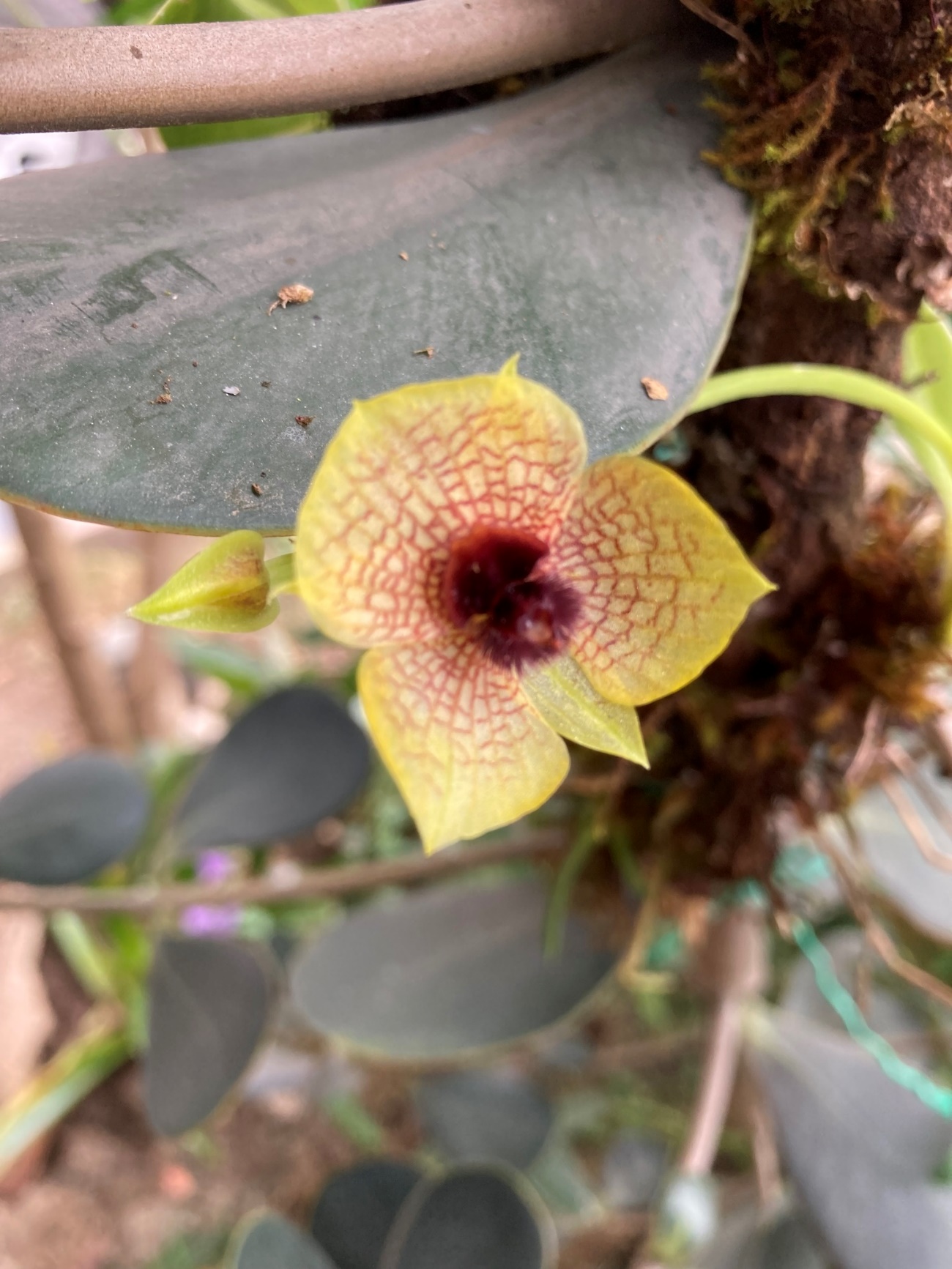


**Supplementary Figure S1.** iNaturalist record of *Telipogon teuscherii* Garay *–* ECUADOR: Azuay, near Cuenca, Aug 2023, *fkarste* <https://www.inaturalist.org/observations/176645191>


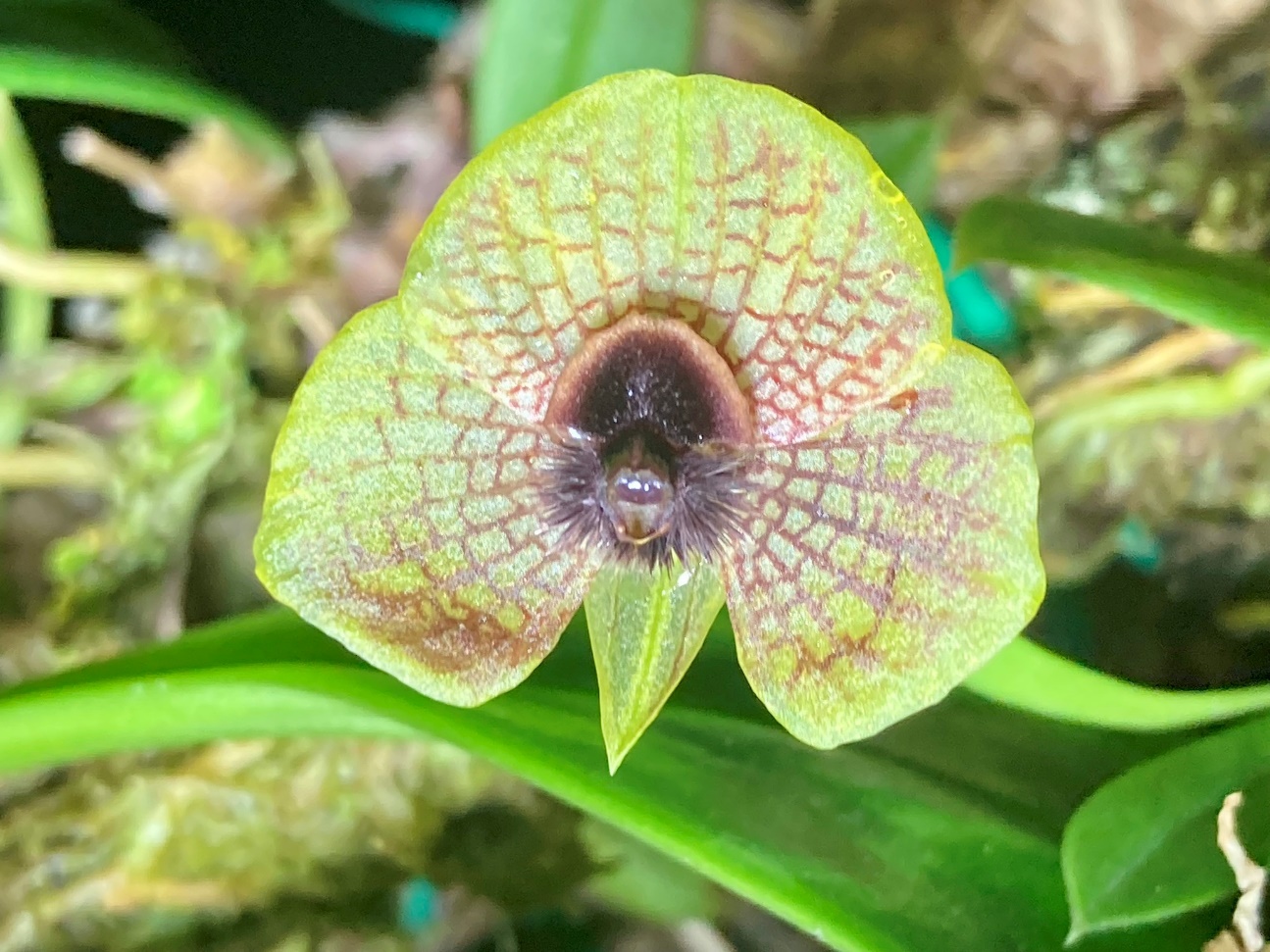


**Supplementary Figure S2.** iNaturalist record of *Telipogon teuscherii* Garay *–* ECUADOR: Azuay, near San Felipe de Molleturo, 30 Jul 2025, *fkarste* <https://www.inaturalist.org/observations/302520547>


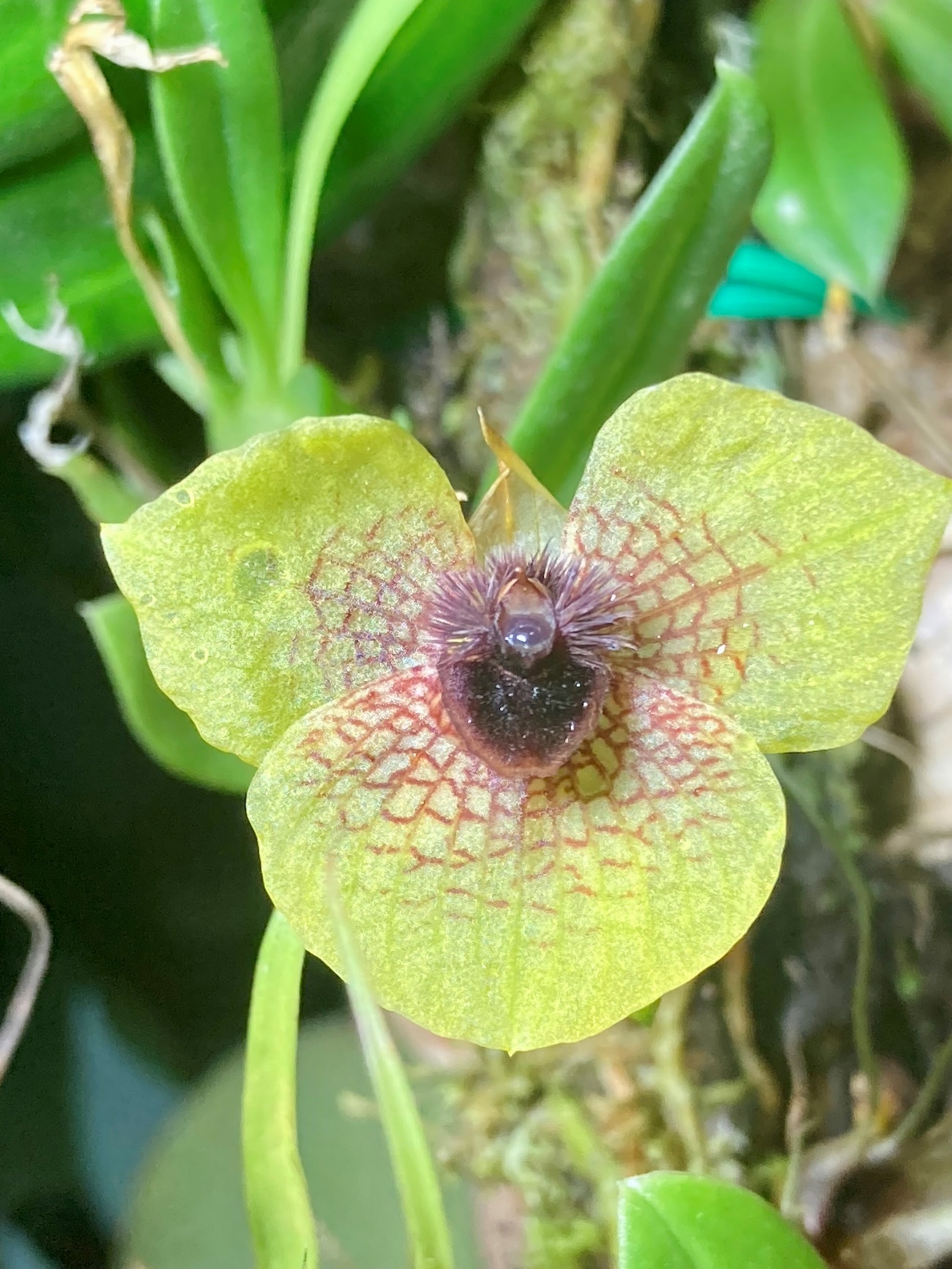


**Supplementary Figure S3.** iNaturalist record of *Telipogon teuscherii* Garay*. –* ECUADOR: Azuay, near San Felipe de Molleturo, 30 Jul 2025, *fkarste* <https://www.inaturalist.org/observations/302520681>
